# Medicare Part D formulary coverage of SGLT2 inhibitors across cardiovascular-kidney-metabolic indications, 2019–2026

**DOI:** 10.1007/s12020-026-04821-2

**Published:** 2026-10-29

**Authors:** Vidur Jain, Rozalina G. McCoy

**Affiliations:** 1 Division of Endocrinology, Diabetes, and Nutrition, Department of Medicine, University of Maryland School of Medicine, 670 West Baltimore Street, Baltimore, MD 21201 USA; 2 University of Maryland Institute for Health Computing, North Bethesda, MD USA

**Keywords:** SGLT2 inhibitors, Medicare Part D, Formulary restrictions, Prior authorization, Inflation Reduction Act

## Abstract

**Purpose:**

Sodium-glucose cotransporter-2 inhibitors (SGLT2i) carry Class I, Level A recommendations for type 2 diabetes (T2D), heart failure (HF), and chronic kidney disease (CKD) but remain underused outside T2D, with formulary restrictions commonly cited as a contributing cause. We assessed whether Medicare Part D formulary restrictions account for this gap and whether the restrictions differ across cardiovascular-kidney-metabolic (CKM) indications.

**Methods:**

Cross-sectional analysis of national Medicare Part D formulary files for plan years 2019–2026, using the January/February snapshot (410 formularies in 2019, 330 in 2026; 13,726 SGLT2i monotherapy entries). We measured entry-level prior authorization (PA) and step-therapy prevalence, the proportion of formularies offering at least one and at least two unrestricted SGLT2i drugs, and tier distribution, across three indication groups reflecting cumulative regulatory approvals.

**Results:**

PA was uncommon across all indications and years, never exceeding 1.6%, and at least 98% of formularies offered a PA-free SGLT2i in every indication group-year. Step therapy fell to zero by 2025 then rebounded in 2026, concentrated on empagliflozin, whose entry-level step therapy rate rose from 0.00% to 18.18%, coincident with the effective date of its negotiated maximum fair price; co-negotiated dapagliflozin showed no comparable increase (1.21%). Tier 3 was the dominant placement (61.9–78.7%). Canagliflozin coverage fell from 58.3% of formularies in 2019 to 5.8% in 2026, and only 5.8% offered two step-free SGLT2i for CKD by 2026.

**Conclusion:**

Formulary restrictions do not appear to be a major barrier to SGLT2i use among Medicare beneficiaries, redirecting attention toward clinician- and system-level factors. Narrowing CKD-indication approved options and an emergent step-therapy rebound warrant monitoring.

**Supplementary Information:**

The online version contains supplementary material available at https://doi.org/10.1007/s12020-026-04821-2.

## Introduction

Guideline-recommended therapies for cardiovascular-kidney-metabolic (CKM) conditions remain substantially underused, leaving many high-risk patients without proven cardiovascular and kidney protection. Sodium-glucose cotransporter-2 inhibitors (SGLT2i) have evolved into foundational CKM therapies with Class I, Level A evidence for heart failure (HF), chronic kidney disease (CKD), and type 2 diabetes (T2D) [1, 2], yet their use outside of T2D remains low. Three agents have received US Food and Drug Administration (FDA) approvals spanning the three CKM indications: canagliflozin (T2D 2013, CKD 2019), dapagliflozin (T2D 2014, HF 2020, CKD 2021), and empagliflozin (T2D 2014, HF 2021, CKD 2023) [3].

Most recent estimates indicate that 6.7 million U.S. adults live with HF, more than 36 million with T2D, and more than 37 million with CKD [4, 5], underscoring the importance of SGLT2i, which have demonstrated benefit for reducing all-cause and cardiovascular mortality, HF hospitalizations, and CKD progression. Yet among patients with a Class 1A indication for SGLT2i, receipt reached only 3.1% in those without diabetes in comparison to 11.9% in those with [6]; prescribing was also higher in health systems serving a lower proportion of Medicare beneficiaries, suggesting Medicare-specific barriers to access.

Whether Medicare Part D formulary restrictions drive this gap is unknown. Prior work focused exclusively on T2D indications, examined formularies before 2023, and predated the drug negotiation provisions of the Inflation Reduction Act (IRA) that likely impacted coverage considerations [7, 8]. The IRA established Medicare drug price negotiation in batches: the first ten drugs were selected in August 2023, with negotiated maximum fair prices effective January 1, 2026, and plans are required to cover selected drugs once these prices take effect. Using Centers for Medicare & Medicaid Services (CMS) Medicare Part D Formulary Files (2019–2026), we address these gaps by characterizing prior authorization (PA), step therapy, and tier placement for SGLT2i across T2D, HF, and CKD indications.

## Materials and methods

We analyzed the CMS Basic Drugs Formulary Files for 2019–2026 using the January/February snapshot for each year; these files include every National Drug Code (NDC) and RxNorm Concept Unique Identifier (RXCUI) for each Medicare Part D formulary, with binary indicators for PA, step therapy, and tier level. Medicare Part D formularies are largely fixed at the start of each year, and CMS restricts mid-year changes that reduce access; the early-year snapshot therefore reflects each plan’s stable formulary design for that specific year. Reporting follows the STROBE guideline for observational studies. Formularies are cross-sectional by design: the same formulary identifier can recur across years under CMS’s coding scheme, and each year was analyzed as an independent snapshot rather than a linked longitudinal panel of the same plans or beneficiaries.

SGLT2i monotherapy products were identified by exact match to 6 RXCUIs representing the 2 marketed dose strengths of each study drug (dapagliflozin, RXCUI 1486977 and 1486981 [Farxiga]; empagliflozin, RXCUI 1545664 and 1545668 [Jardiance]; canagliflozin, RXCUI 1373469 and 1373473 [Invokana]), matched against the RXCUI field reported natively in each CMS formulary file; ertugliflozin and bexagliflozin were excluded (no CKD or HF indication). Because fixed-dose combination products (e.g., dapagliflozin-metformin, empagliflozin-linagliptin) carry distinct RXCUIs from these 6 monotherapy products, they were structurally excluded by this allowlist and required no separate filtering step; their formulary placement reflects the combined product rather than the SGLT2i component alone, and they carry different indications from the monotherapy agents. This yielded six drug-strength products (two strengths per drug) and a final analytic file of 13,726 entries (2019–2026) with no missing values for formulary identifier, RXCUI, NDC, or tier, and no exact duplicate formulary-drug-year rows. RxNorm matching, rather than NDC label prefixes, avoids contamination with unrelated drugs from the same manufacturer. Each formulary year was evaluated across three indication groups reflecting cumulative FDA approvals: T2D (all drugs, all years), HF (dapagliflozin 2021–2026, empagliflozin 2022–2026), and CKD (canagliflozin 2020–2026, dapagliflozin 2022–2026). These groups reflect the FDA-approved eligible population for each drug-year rather than clinical risk stratification; because Medicare Part D formulary files record utilization management (e.g., PA, step therapy) at the level of the drug product rather than the patient’s indication or cardiovascular risk profile, a formulary cannot apply different restrictions to a beneficiary based on, for example, established atherosclerotic cardiovascular disease or high cardiovascular risk within the T2D group; our T2D estimates therefore describe coverage available to all beneficiaries with T2D rather than the risk-stratified subgroups for whom SGLT2i carry the strongest guideline recommendation.

We report 2 complementary units of analysis throughout the Results: entry-level estimates, whose denominator is the total number of formulary-product listings for that indication-year (e.g., 6,508 empagliflozin entries across the study period), and formulary-level estimates, whose denominator is the number of formularies offering at least one SGLT2i for that indication in that year; the applicable denominator is indicated at first use in each Results subsection. We computed entry-level PA and step-therapy prevalence; formulary-level proportions with at least one and at least two unrestricted SGLT2i (to assess patient-relevant formulary coverage); and tier distributions. Analyses used two units of observation. Entry-level analyses treat each formulary-product listing (a drug-strength combination within a formulary-year) as the unit. Drug-formulary-level analyses assign each drug its lowest listed tier within each formulary-year, so that each drug contributes once per formulary. Because RxNorm matching yielded exactly two strengths per drug, each drug contributed exactly two entries per formulary-year in every study year (mean, median, and maximum), and both strengths were consistently placed on the same tier; entry-level and drug-formulary-level tier distributions were therefore identical, indicating that entry-level percentages are not biased by differences in the number of listed products across manufacturers. Entries with tier values outside 1–5 (270 entries, 2.0%, all recorded as tier 6) were excluded from tier analyses.

All analyses were descriptive. This study analyzed publicly available, de-identified administrative data describing Medicare Part D plan formulary design. It involved no human participants, no individual-level health information, and no intervention; therefore, it did not require institutional review board approval or informed consent.

## Results

The number of distinct Medicare Part D formularies rose from 410 formularies in 2019 to a peak of 456 in 2022, then declined to 330 by 2026. The RxNorm filter yielded 13,726 SGLT2i formulary entries (empagliflozin 6,508; dapagliflozin 4,988; and canagliflozin 2,230).

**Fig. 1:**
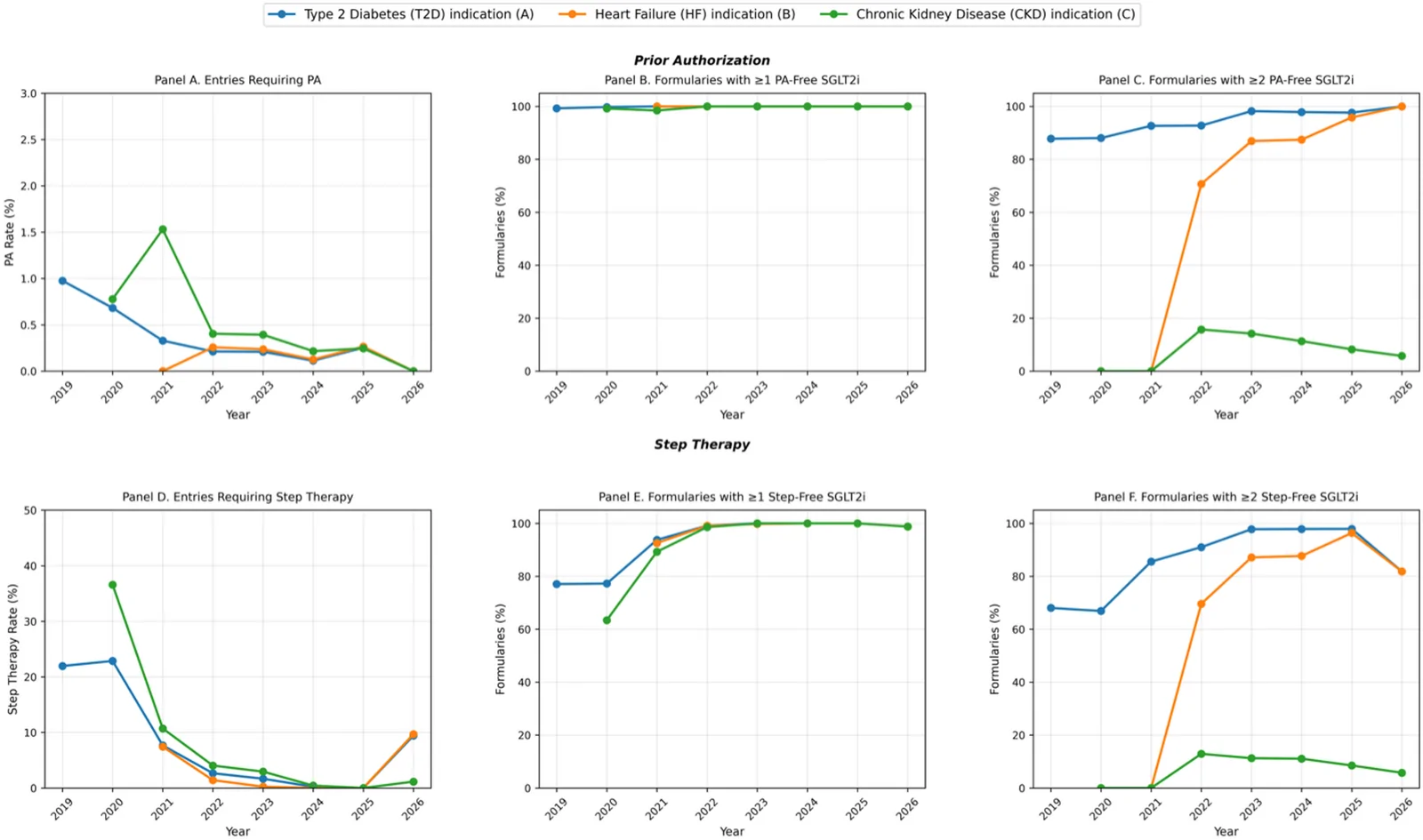
Prior authorization and step therapy burden on SGLT2i monotherapy in Medicare Part D plans, 2019–2026. **A** Proportion of formulary entries (drug-strength listings) requiring prior authorization; **B** proportion of Medicare Part D formularies offering at least one prior-authorization-free SGLT2i; **C** proportion offering two or more prior-authorization-free SGLT2i; **D**-**F** the parallel step-therapy metrics. Within each panel, lines denote the indication group, which the figure key labels (**A**), (**B**), and (**C**); these parenthetical letters identify indication groups and are distinct from the panel letters. Indication groups are type 2 diabetes (all SGLT2i), heart failure (dapagliflozin from 2021, empagliflozin from 2022), and chronic kidney disease (canagliflozin from 2020, dapagliflozin from 2022)

Entry-level PAs were uncommon. For T2D, PA prevalence declined from 0.98% (2019) to 0.21% (2022) and 0% (2026); for HF, it remained consistently at or below 0.27%; and for CKD, it peaked at 1.53% (95% CI, 0.70–3.30%) in 2021 but declined to at or below 0.40% thereafter (Fig. 1A; full annual estimates with 95% CIs for all measures and indication groups are provided in Supplementary Table S1). At least one PA-free SGLT2i was offered by at least 98% of formularies in every indication group-year (Fig. 1B). The proportion offering at least two PA-free SGLT2i rose from 87.8% (2019) to 100% (2026) for T2D, and from 70.7% in 2022 (the first year when two HF-indicated drugs were available) to 100% (2026) for HF. In contrast, only 15.7% of formularies offered two PA-free SGLT2i for CKD in 2022, declining to 5.8% (95% CI, 3.72–8.82%) by 2026 (Fig. 1C).

Entry-level step therapy declined throughout the study period, then reversed in 2026. In the T2D indication, the prevalence of step therapy declined from 21.95% (2019) to 0.00% (2025) and rose again to 9.43% (2026) (Fig. 1D). Similar patterns were seen for HF (7.43% to 0.00% to 9.70%) and CKD indications. The proportion of formularies with at least one step-free SGLT2i exceeded 98% by 2023 in all groups (Fig. 1E); the proportion with at least two step-free SGLT2i peaked near 98% in T2D (2024–2025, 97.9%-97.9%) and in HF (2025, 96.4%), before falling to approximately 82% in both groups by 2026, while in the CKD group it never exceeded 12.9% (95% CI, 10.05–16.43%; 2022) and declined to 5.8% (95% CI, 3.72–8.82%) by 2026 (Fig. 1F).

**Fig. 2:**
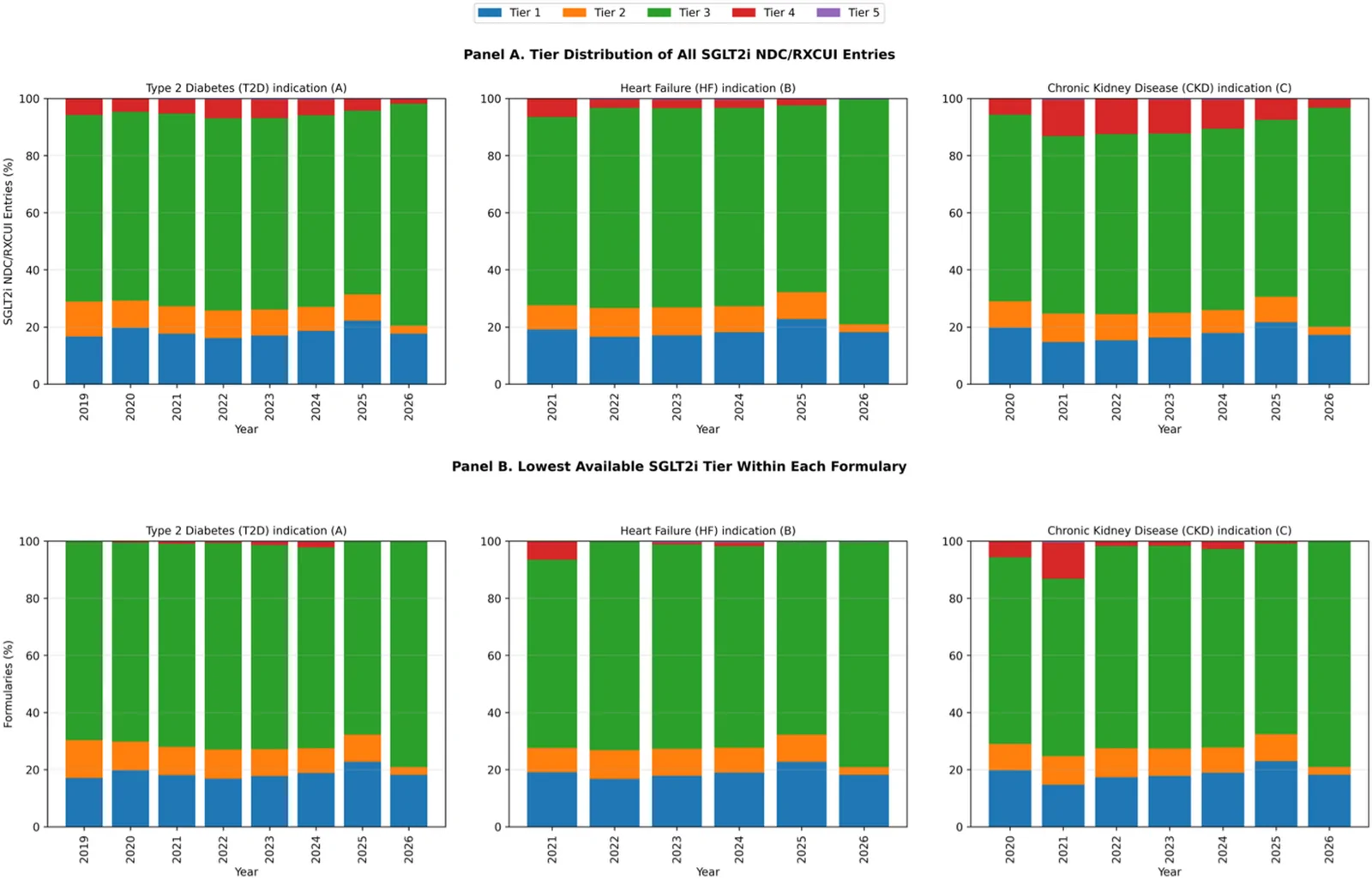
SGLT2i tier placement on Medicare Part D formularies, 2019–2026. **A** Tier distribution of all SGLT2i formulary entries (drug-strength listings) within each indication group; **B** distribution of the lowest available SGLT2i tier within each formulary. Indication groups are defined as in Fig. 1. While tier designations within each plan may vary, Tier 1 generally represents preferred generic drugs; Tier 2, other sgeneric drugs; Tier 3, preferred brand drugs; Tier 4, non-preferred drugs; and Tier 5, highest-cost specialty drugs

Tier 3 (preferred brand) was the dominant placement, accounting for 61.9–78.7% of entries across all indication groups and years. Tiers 1–2 accounted for approximately 20–32% of entries, and Tier 4 was uncommon (at or below 6.6% in the T2D and HF groups), with a modestly higher prevalence in the CKD group (peak 12.6% in 2021). Tier 5 placement was negligible (at or below 0.5% in all group-years). Aggregating to the lowest tier per drug within each formulary produced identical distributions (Fig. 2A). The lowest tier available within each formulary across all indication-eligible agents is shown in Fig. 2B.

Formulary coverage diverged sharply across all three agents. Dapagliflozin coverage rose from 46.8% of formularies (2019) to 100% (2026), and empagliflozin rose from 94.9% to 100%, whereas canagliflozin declined (58.3% to 5.8%). The 2026 step-therapy rebound was concentrated almost entirely on empagliflozin, whose entry-level step-therapy rate rose from 0.00% (0 of 1,532 entries; 95% CI, 0.00-0.25%) in 2025 to 18.18% (240 of 1,320 entries; 95% CI, 16.19–20.35%) in 2026; dapagliflozin showed no comparable increase (24 of 1,980 entries, 1.21%; 95% CI, 0.82–1.80%, in 2026), and canagliflozin step therapy had fallen to 0% by 2025.

## Discussion

Despite concerns that gaps in SGLT2i use across CKM indications—and particularly for HF and CKD—were due to insurance-mediated barriers, we found that across eight years of Medicare Part D formulary data spanning all major SGLT2i indication expansions, PA requirements were rare and declining, step therapy initially declined to near zero but rebounded across indications in 2026, and Tier 3 (preferred brand) was the most common placement. Tier 5 in Medicare Part D is conventionally reserved for specialty and high-cost products. As preferred oral brand agents, SGLT2i are expected to occupy Tier 3, consistent with our findings.

Our data suggest PA burden is not a meaningful formulary barrier to SGLT2i coverage (unlike for other CKM therapies such as glucagon-like peptide-1 receptor agonists): entry-level PA stayed below 1.6% across all indications (95% CI upper bounds below 3.3% in every group-year; Supplementary Table S1), with at least 98% of formularies offering at least one PA-free SGLT2i every year. Step therapy was likewise low throughout but rebounded in 2026, driven almost entirely by empagliflozin, whose entry-level step-therapy prevalence rose from 0.00% (0 of 1,532 entries; 95% CI, 0.00-0.25%) in 2025 to 18.18% (240 of 1,320 entries; 95% CI, 16.19–20.35%) in 2026, coincident with the January 2026 effective date of its IRA-negotiated maximum fair price. Notably, dapagliflozin, also price-negotiated, showed no comparable increase (1.21%; 95% CI, 0.82–1.80%) [9]. The 2026 step-therapy rebound reflects a tension within the IRA, mandating coverage of negotiated drugs but permitting utilization management restrictions within that requirement. Both empagliflozin and dapagliflozin were among the first ten drugs selected for negotiation, with maximum fair prices effective on January 1, 2026. This descriptive, cross-sectional design cannot establish that the negotiated price caused the empagliflozin-specific increase, and we lacked data on plan rebate agreements, formulary decision-making, or comparator drug economics that would be needed to test this directly; reduced post-negotiation manufacturer rebates are one plausible hypothesis for future work, alongside other possible explanations such as routine annual formulary revision unrelated to negotiation. Because both empagliflozin and dapagliflozin were covered by all formularies in 2026, the concentration of step therapy on empagliflozin reflects a formulary-level requirement, in the plans imposing it, that dapagliflozin be tried before empagliflozin is covered without restriction; how this formulary requirement translates into realized first-line prescribing or treatment patterns cannot be assessed with these plan-level data. Whether this early step-therapy rebound persists and broadens as negotiations expand warrants ongoing surveillance; sustained or growing restrictions on negotiated drugs would help determine whether safeguards in utilization management are needed.

Our findings extend two prior analyses of Medicare Part D coverage for SGLT2 inhibitors. Luo et al. characterized coverage, formulary restrictions, and out-of-pocket costs in 2020 [8], and Wisniewski et al. examined formulary restrictions for SGLT2i and glucagon-like peptide-1 receptor agonists in T2D from 2019 to 2023 [7]. Both were confined to the T2D indication and predated the IRA. Our results are consistent with theirs in showing that prior authorization is applied sparingly to this class relative to other cardiometabolic agents. We extend that observation across the HF and CKD indications as they were approved, and into the first year in which negotiated maximum fair prices were in effect.

Second, the HF-CKD coverage disparity reflects an asymmetric collapse in canagliflozin coverage from 58.3% (2019) to 5.8% (2026), despite its first-in-class CKD indication (September 2019) [10]. One hypothesis is that the 2017 boxed warning for lower-limb amputation, ultimately removed in August 2020 [3], produced a commercial response that persisted beyond its clinical relevance; by 2026, most Part D formularies covering CKD-indicated SGLT2i listed only dapagliflozin among the 3 agents. Coverage nonetheless continued to decline in every subsequent year, falling from 57.9% in 2020 to 21.3% in 2024 and 9.6% in 2025. Our formulary data cannot adjudicate between the boxed-warning hypothesis and other possible explanations, including changes in manufacturer rebate contracting or broader formulary consolidation over the study period.

Our study’s strengths include complete national formulary coverage and eight years of data spanning all three CKM indications, including the post-IRA period. Limitations include the descriptive design. Our data describe formulary design—whether a drug is listed, and under what utilization management—and do not include beneficiary cost-sharing (copayment or coinsurance amounts) or beneficiary-level utilization data; a drug listed without prior authorization may still be unaffordable at its assigned cost-sharing tier, and formulary coverage does not guarantee that eligible patients are ultimately prescribed it or fill the medication, so our findings should be interpreted as describing plan-level coverage design rather than realized patient access, utilization, or affordability. Prior authorization and step therapy are recorded as binary indicators, capturing the presence of utilization management but not its nuance. We excluded ertugliflozin (no HF/CKD indication or demonstrated cardiovascular benefit) and bexagliflozin (minimally covered), which is unlikely to affect class-level estimates. Finally, 2026 data reflect the January/February snapshot—weeks after negotiated prices took effect—and may not capture the plans’ full response. The unit of analysis is the formulary rather than the individual, so the data contain no beneficiary-level characteristics, and sex- and race/ethnicity-based differences in coverage could not be assessed.

Across eight years of national Medicare Part D formulary data spanning all three SGLT2i cardiovascular-kidney-metabolic indications, prior authorization was rare and declining, and nearly all formularies offered at least one unrestricted SGLT2i in every indication group and year. Formulary design does not appear to be the primary barrier to SGLT2i use in this population; closing the prescribing gap will require targeting clinician inertia across specialty boundaries, fragmented cardiology-nephrology-primary care coordination, and patient initiation and persistence on therapy. Two developments warrant continued surveillance: the narrowing of unrestricted options for CKD following the collapse of canagliflozin coverage, and the 2026 rebound in step therapy concentrated on empagliflozin.

## Supplementary information

Below is the link to the electronic supplementary material.

Supplementary Material 1

## Data Availability

The CMS Medicare Part D Basic Drugs Formulary files analyzed in this study are publicly available as part of the Monthly Prescription Drug Plan Formulary and Pharmacy Network Information Public Use Files from the Centers for Medicare & Medicaid Services (https://data.cms.gov). Analytic code is available from the corresponding author upon reasonable request.
