# Supplementary material for: Medicare Part D formulary coverage of SGLT2 inhibitors across cardiovascular-kidney-metabolic indications, 2019–2026
Source: Endocrine. 2026 Oct 29;91(1):359. doi: 10.1007/s12020-026-04821-2 (PMC13719726; doi:10.1007/s12020-026-04821-2)
Supplement: Supplementary file 1 [file 12020_2026_4821_MOESM1_ESM.docx]

**Supplementary Table S1. Annual entry-level and formulary-level prior authorization (PA) and step-therapy estimates, by indication group, 2019–2026, with 95% Wilson score confidence intervals.**

| **Year** | **Group** | **N**  **entries** | **PA, n /**  **% (95% CI)** | **Formu-**  **laries** | **≥1 PA-free,**  **n / % (95% CI)** | **≥2 PA-free,**  **n / % (95% CI)** | **STEP, n /**  **% (95% CI)** | **≥1 STEP-free,**  **n / % (95% CI)** | **≥2 STEP-free,**  **n / % (95% CI)** |
| --- | --- | --- | --- | --- | --- | --- | --- | --- | --- |
| 2019 | T2D | 1640 | 16 / 0.98 (0.60–1.58) | 410 | 407 / 99.27 (97.87–99.75) | 360 / 87.80 (84.28–90.63) | 360 / 21.95 (20.01–24.02) | 316 / 77.07 (72.76–80.88) | 279 / 68.05 (63.39–72.38) |
| 2020 | T2D | 1758 | 12 / 0.68 (0.39–1.19) | 444 | 443 / 99.77 (98.74–99.96) | 391 / 88.06 (84.72–90.76) | 402 / 22.87 (20.96–24.89) | 343 / 77.25 (73.13–80.91) | 297 / 66.89 (62.39–71.11) |
| 2021 | T2D | 1826 | 6 / 0.33 (0.15–0.72) | 450 | 450 / 100.00 (99.15–100.00) | 417 / 92.67 (89.88–94.73) | 140 / 7.67 (6.53–8.98) | 422 / 93.78 (91.15–95.66) | 385 / 85.56 (82.01–88.50) |
| 2022 | T2D | 1884 | 4 / 0.21 (0.08–0.54) | 456 | 456 / 100.00 (99.16–100.00) | 423 / 92.76 (90.01–94.80) | 50 / 2.65 (2.02–3.48) | 452 / 99.12 (97.77–99.66) | 415 / 91.01 (88.03–93.30) |
| 2023 | T2D | 1914 | 4 / 0.21 (0.08–0.54) | 451 | 451 / 100.00 (99.16–100.00) | 443 / 98.23 (96.54–99.10) | 32 / 1.67 (1.19–2.35) | 451 / 100.00 (99.16–100.00) | 441 / 97.78 (95.97–98.79) |
| 2024 | T2D | 1764 | 2 / 0.11 (0.03–0.41) | 422 | 422 / 100.00 (99.10–100.00) | 413 / 97.87 (96.00–98.87) | 4 / 0.23 (0.09–0.58) | 422 / 100.00 (99.10–100.00) | 413 / 97.87 (96.00–98.87) |
| 2025 | T2D | 1582 | 4 / 0.25 (0.10–0.65) | 384 | 384 / 100.00 (99.01–100.00) | 375 / 97.66 (95.61–98.76) | 0 / 0.00 (0.00–0.24) | 384 / 100.00 (99.01–100.00) | 376 / 97.92 (95.94–98.94) |
| 2026 | T2D | 1358 | 0 / 0.00 (0.00–0.28) | 330 | 330 / 100.00 (98.85–100.00) | 330 / 100.00 (98.85–100.00) | 128 / 9.43 (7.98–11.10) | 326 / 98.79 (96.93–99.53) | 270 / 81.82 (77.30–85.61) |
| 2021 | HF | 592 | 0 / 0.00 (0.00–0.64) | 296 | 296 / 100.00 (98.72–100.00) | 0 / 0.00 (0.00–1.28) | 44 / 7.43 (5.58–9.83) | 274 / 92.57 (89.00–95.04) | 0 / 0.00 (0.00–1.28) |
| 2022 | HF | 1554 | 4 / 0.26 (0.10–0.66) | 454 | 454 / 100.00 (99.16–100.00) | 321 / 70.70 (66.36–74.70) | 22 / 1.42 (0.94–2.13) | 450 / 99.12 (97.76–99.66) | 316 / 69.60 (65.22–73.66) |
| 2023 | HF | 1690 | 4 / 0.24 (0.09–0.61) | 451 | 451 / 100.00 (99.16–100.00) | 392 / 86.92 (83.49–89.72) | 4 / 0.24 (0.09–0.61) | 450 / 99.78 (98.75–99.96) | 393 / 87.14 (83.73–89.92) |
| 2024 | HF | 1584 | 2 / 0.13 (0.03–0.46) | 422 | 422 / 100.00 (99.10–100.00) | 369 / 87.44 (83.94–90.27) | 0 / 0.00 (0.00–0.24) | 422 / 100.00 (99.10–100.00) | 370 / 87.68 (84.20–90.48) |
| 2025 | HF | 1508 | 4 / 0.27 (0.10–0.68) | 384 | 384 / 100.00 (99.01–100.00) | 368 / 95.83 (93.34–97.42) | 0 / 0.00 (0.00–0.25) | 384 / 100.00 (99.01–100.00) | 370 / 96.35 (93.97–97.82) |
| 2026 | HF | 1320 | 0 / 0.00 (0.00–0.29) | 330 | 330 / 100.00 (98.85–100.00) | 330 / 100.00 (98.85–100.00) | 128 / 9.70 (8.22–11.41) | 326 / 98.79 (96.93–99.53) | 270 / 81.82 (77.30–85.61) |
| 2020 | CKD | 514 | 4 / 0.78 (0.30–1.98) | 257 | 255 / 99.22 (97.21–99.79) | 0 / 0.00 (0.00–1.47) | 188 / 36.58 (32.53–40.83) | 163 / 63.42 (57.38–69.07) | 0 / 0.00 (0.00–1.47) |
| 2021 | CKD | 392 | 6 / 1.53 (0.70–3.30) | 196 | 193 / 98.47 (95.60–99.48) | 0 / 0.00 (0.00–1.92) | 42 / 10.71 (8.02–14.17) | 175 / 89.29 (84.18–92.88) | 0 / 0.00 (0.00–1.92) |
| 2022 | CKD | 990 | 4 / 0.40 (0.16–1.03) | 426 | 426 / 100.00 (99.11–100.00) | 67 / 15.73 (12.58–19.49) | 40 / 4.04 (2.98–5.46) | 420 / 98.59 (96.96–99.35) | 55 / 12.91 (10.05–16.43) |
| 2023 | CKD | 1018 | 4 / 0.39 (0.15–1.01) | 444 | 444 / 100.00 (99.14–100.00) | 63 / 14.19 (11.25–17.74) | 30 / 2.95 (2.07–4.18) | 444 / 100.00 (99.14–100.00) | 50 / 11.26 (8.65–14.54) |
| 2024 | CKD | 926 | 2 / 0.22 (0.06–0.78) | 415 | 415 / 100.00 (99.08–100.00) | 47 / 11.33 (8.62–14.74) | 4 / 0.43 (0.17–1.11) | 415 / 100.00 (99.08–100.00) | 46 / 11.08 (8.41–14.47) |
| 2025 | CKD | 816 | 2 / 0.25 (0.07–0.89) | 376 | 376 / 100.00 (98.99–100.00) | 31 / 8.24 (5.87–11.47) | 0 / 0.00 (0.00–0.47) | 376 / 100.00 (98.99–100.00) | 32 / 8.51 (6.09–11.77) |
| 2026 | CKD | 698 | 0 / 0.00 (0.00–0.55) | 330 | 330 / 100.00 (98.85–100.00) | 19 / 5.76 (3.72–8.82) | 8 / 1.15 (0.58–2.25) | 326 / 98.79 (96.93–99.53) | 19 / 5.76 (3.72–8.82) |

*Group indicates the cardiovascular-kidney-metabolic indication group (T2D, type 2 diabetes; HF, heart failure; CKD, chronic kidney disease), reflecting the FDA-approved eligible population for each drug-year (see Materials and methods). N entries and Formularies are the entry-level and formulary-level denominators, respectively, shared across the corresponding PA and step-therapy (STEP) columns. Each remaining cell reports the numerator (n), followed by the percentage and 95% Wilson score confidence interval in parentheses; Wilson intervals are appropriate near proportions of 0% or 100%. A numerator of 0 with a valid nonzero denominator reflects a true zero (eg, no formulary offered 2 or more step-free CKD-indicated SGLT2i in 2020 or 2021), not a missing value.*
